# Jugular Vein Intracardiac Echocardiography-Guided Percutaneous Transluminal Septal Myocardial Ablation: A Novel Approach in 4 Cases

**DOI:** 10.1016/j.jaccas.2025.103307

**Published:** 2025-03-12

**Authors:** Hiroto Yagasaki, Keitaro Watanabe, Takeki Suzuki, Makoto Iwama, Shinichiro Tanaka, Toshiyuki Noda

**Affiliations:** a Department of Cardiology, Gifu Prefectural General Medical Center, Gifu, Japan; b Department of Medicine, Indiana University School of Medicine, Indianapolis, Indiana, USA; c Department of Cardiology, Asahi University Hospital, Gifu City, Gifu, Japan

**Keywords:** cardiomyopathy, echocardiography, treatment

## Abstract

**Objective:**

This study sought to describe a novel technique for percutaneous transluminal septal myocardial ablation using intracardiac echocardiography (ICE) through the right internal jugular vein (JV) in patients with hypertrophic obstructive cardiomyopathy.

**Key Steps:**

Prepare the equipment and establish vascular access, insert the JV ICE catheter using a sterile sleeve, perform initial screening with JV ICE to obtain key cardiac views, identify target septal branch using coronary angiography and JV ICE, monitor contrast injection and ethanol administration in real time with JV ICE, continuously assess for immediate complications, and evaluate postprocedure gradient reduction and ablation efficacy.

**Potential Pitfalls:**

Risks include vascular complications, arrhythmias, cardiac tamponade, embolism, and infections. JV ICE requires specific skills, presenting a learning curve. Mitigation strategies involve ultrasound-guided access, careful catheter manipulation, strict asepsis, comprehensive training, optimized settings, and continuous monitoring.

## Graphical Abstract

**Figure:**
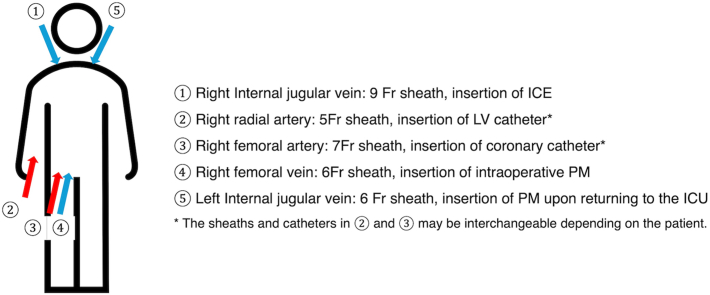

Hypertrophic cardiomyopathy (HCM) is a genetic disorder affecting 1 in 500 people and characterized by left ventricular (LV) wall thickening.^1^ Approximately 70% of patients with HCM develop hypertrophic obstructive cardiomyopathy (HOCM) with LV outflow tract obstruction and symptoms.^2^ Percutaneous transluminal septal myocardial ablation (PTSMA) is a treatment for symptomatic HOCM, used in drug-refractory cases or when surgery is contraindicated.^1^ Transthoracic echocardiography (TTE) with myocardial contrast echocardiography traditionally guides PTSMA.^3^^,^^4^ Alternative imaging techniques, such as transesophageal echocardiography (TEE) or ICE through the femoral vein (FV), are also used in some cases.^5^^,^^6^ However, these methods have limitations. This report presents PTSMA cases using a novel approach: ICE through the right internal jugular vein (JV), which may address some of limitations associated with TTE, TEE, and FV ICE approaches.

**Take-Home Messages**

• Echocardiographic evaluation during PTSMA, traditionally performed using TTE, TEE, or FV ICE, can potentially include JV ICE as an alternative for guiding and monitoring the procedure.
• JV ICE-guided PTSMA could offer enhanced visualization and real-time monitoring, thus potentially improving safety and efficacy during the procedure compared with traditional approaches.

## Case Summary

This section presents 4 cases of JV ICE-guided PTSMA, highlighting key procedural aspects, challenges, and outcomes. The patients gave written informed consent for the publication of their case reports and accompanying images.

### Case 1

**Figure 1:**
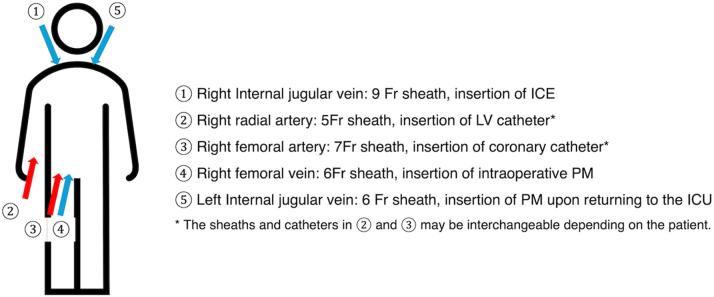
Setup Example ICE = intracardiac echocardiography; ICU = intensive care unit; LV = left ventricular; PM = pacemaker.

A 78-year-old man with HOCM presented with syncope and falls. After arrhythmias and epilepsy were ruled out, TTE revealed an LV outflow tract gradient of 85 mm Hg. PTSMA was performed with the patient under general anesthesia. Vascular access was established as shown in Figure 1, with an 8-F AcuNav ICE catheter (Siemens) advanced from the right internal JV. A sterile sleeve over the ICE catheter allowed nonsterile manipulation while maintaining field sterility.

**Figure 2:**
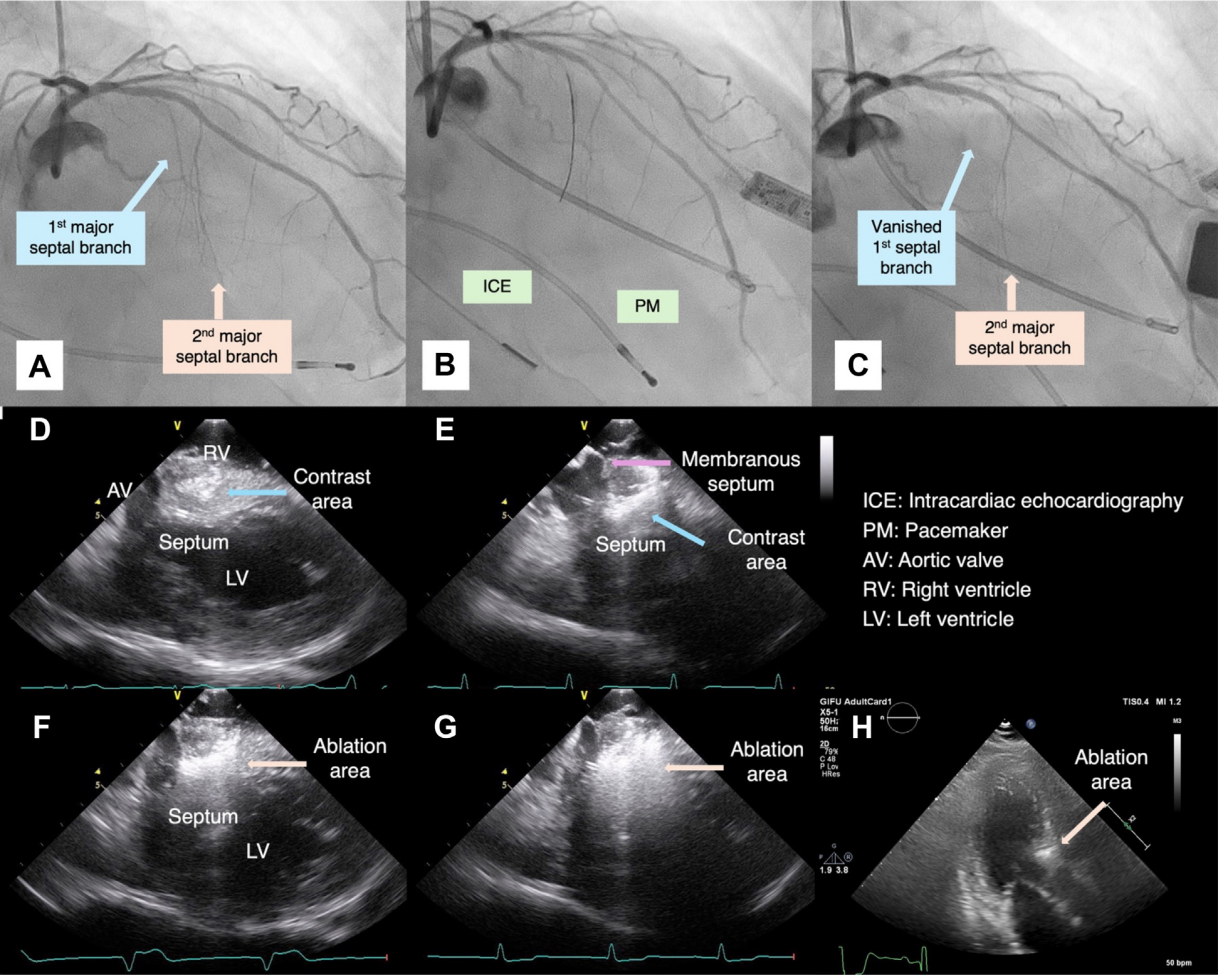
Imaging in Case 1 (A to C) Coronary angiography (A) before ablation, (B) during ballooning, and (C) post ablation. (D to G) Intracardiac echocardiography (ICE) (D) in the early phase of contrast injection, (E) in the late phase of contrast injection, (F) in the early phase of ethanol injection, and (G) in the late phase of ethanol injection. (H) A 3-chamber view by myocardial contrast echocardiography using transthoracic echocardiography. The contrast agent initially stains the myocardium in a wedge-shaped pattern from (D) the right ventricular side, eventually reaching (E) the surface of the left ventricle (LV). (F and G) The myocardium is gradually stained in proportion to the amount of alcohol injected. Intracardiac echocardiography provides superior visualization of the ablated myocardium compared with (H) transthoracic echocardiography. AV = aortic valve; PM = pacemaker; RV = right ventricle.

Contrast agent injection into the first major septal branch from the left anterior descending (LAD) artery identified the target area (Figures 2A, 2B, 2D, and 2E, Video 1). Ethanol (2 mL) was administered over approximately 8 minutes, causing transient complete atrioventricular block (cAVB). ICE, coronary angiography, and TTE confirmed successful ablation (Figures 2C and 2F to 2H, Video 2). A temporary pacemaker (PM) was placed through the left internal JV (Figure 1). The patient was discharged without complications 7 days post procedure. At 6months, symptoms recurred (gradient, 100 mm Hg), necessitating a repeat PTSMA (Table 1). The second procedure targeted the second major septal branch and reduced the gradient to <50 mm Hg, as well as improving symptoms.

**Table 1:** Summary of Patient Clinical Characteristics, Changes in Laboratory Data, Pressure Gradient, and Symptoms

Patient #	Age, y	Sex	Procedure Time, min	Target Vessel	Alcohol Volume, mL	CK, IU/L	CK-MB, IU/L	Preprocedure			6 Months Postprocedure			Complication	Second Session
								Rest Gradient, mm Hg	Valsalva Gradient, mm Hg	Symptom	Rest Gradient, mm Hg	Valsalva Gradient, mm Hg	Symptom		
1	78	M	216	LAD	2	808	−	85	101	Dyspnea	33	100	+	−	+
2	75	F	247	HL → LAD	2	898	187	102	−	Dyspnea	75^a^	120^a^	+	−	+
3	78	F	177	LAD → HL	1	484	79	44	98	Dyspnea	13	13	−	Complete AV block → leadless pacemaker	−
4	79	F	167	LAD	2	2,777	462	67	−	Dyspnea	5	−	−	−	−

AV = atrioventricular; CK = creatine kinase; CK-MB = creatine kinase MB isoenzyme; HL = high lateral branch (coronary artery); LAD = left anterior descending (coronary artery); Pt = patient; + = positive; − = negative.

a At 3 months.

### Case 2

**Figure 3:**
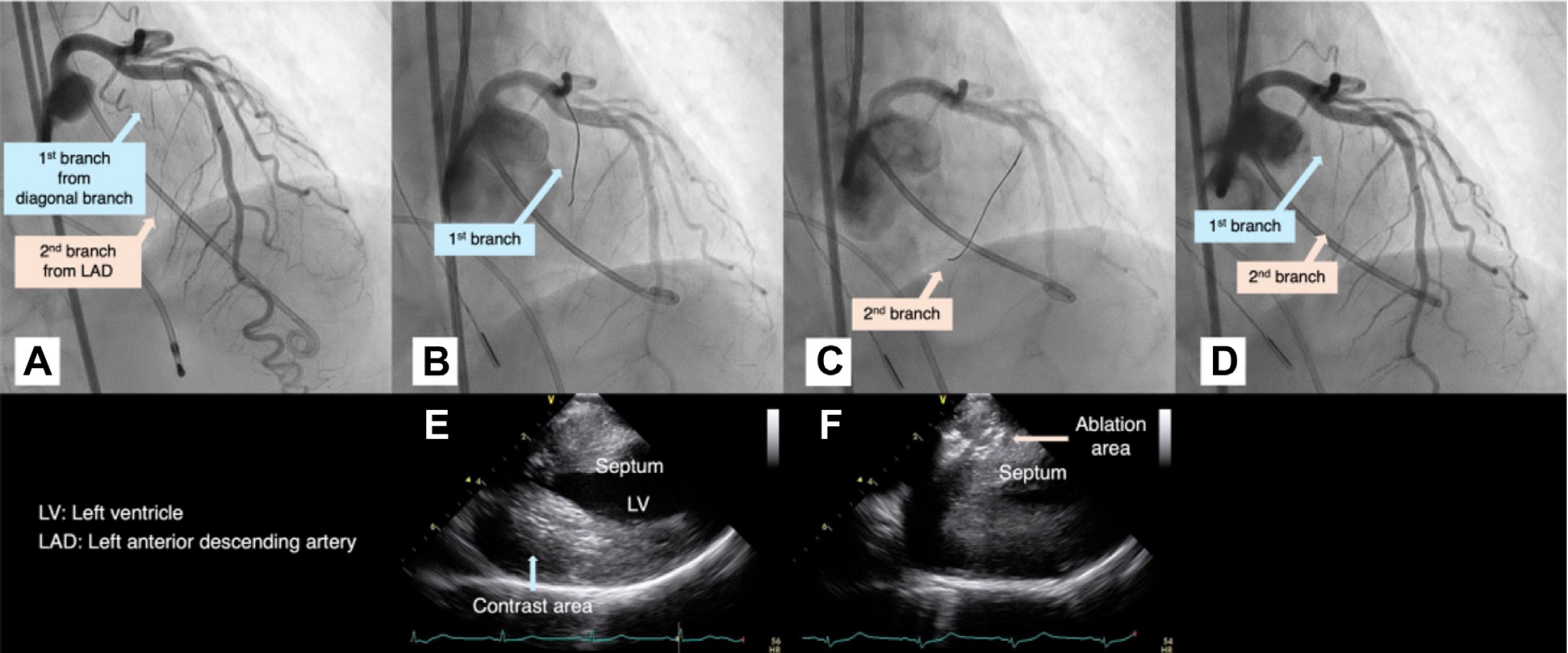
Imaging in Case 2, Second Session (A to D) Coronary angiography (A) before ablation, (B) during wiring to the first branch from the diagonal branch, (C) during wiring to the second branch from the left anterior descending (LAD) artery, and (D) post ablation. (E and F) Jugular vein intracardiac echocardiography showing (E) contrast agent injection to the first branch from the diagonal branch and (F) ethanol injection to the second branch from the left anterior descending artery. High signal intensity is observed up to the membranous septal region. LV = left ventricle.

A 75-year-old woman undergoing preoperative evaluation for left lung adenocarcinoma presented with worsening exertional dyspnea. Assessment revealed HOCM (gradient, 103 mm Hg). PTSMA was performed before the lung cancer surgery, using the same setup as in case 1. Angiography showed a branch near the interventricular septum from the diagonal branch (Figures 3A and 3B). ICE-guided contrast agent injection revealed unsuitable basal inferolateral wall opacification (Figure 3E, Video 3). A septal branch from the LAD artery was then targeted (Figure 3C). Ethanol injection followed the method used in case 1 (Figure 3F, Video 3). The procedure was terminated early even though the ablated branch remained patent, to avoid complications from excessive ethanol use (Figure 3D). At 3 months, with persistent symptoms (gradient, 75 mm Hg), repeat PTSMA was performed successfully (Table 1). The patient subsequently underwent lung cancer surgery.

### Case 3

**Figure 4:**
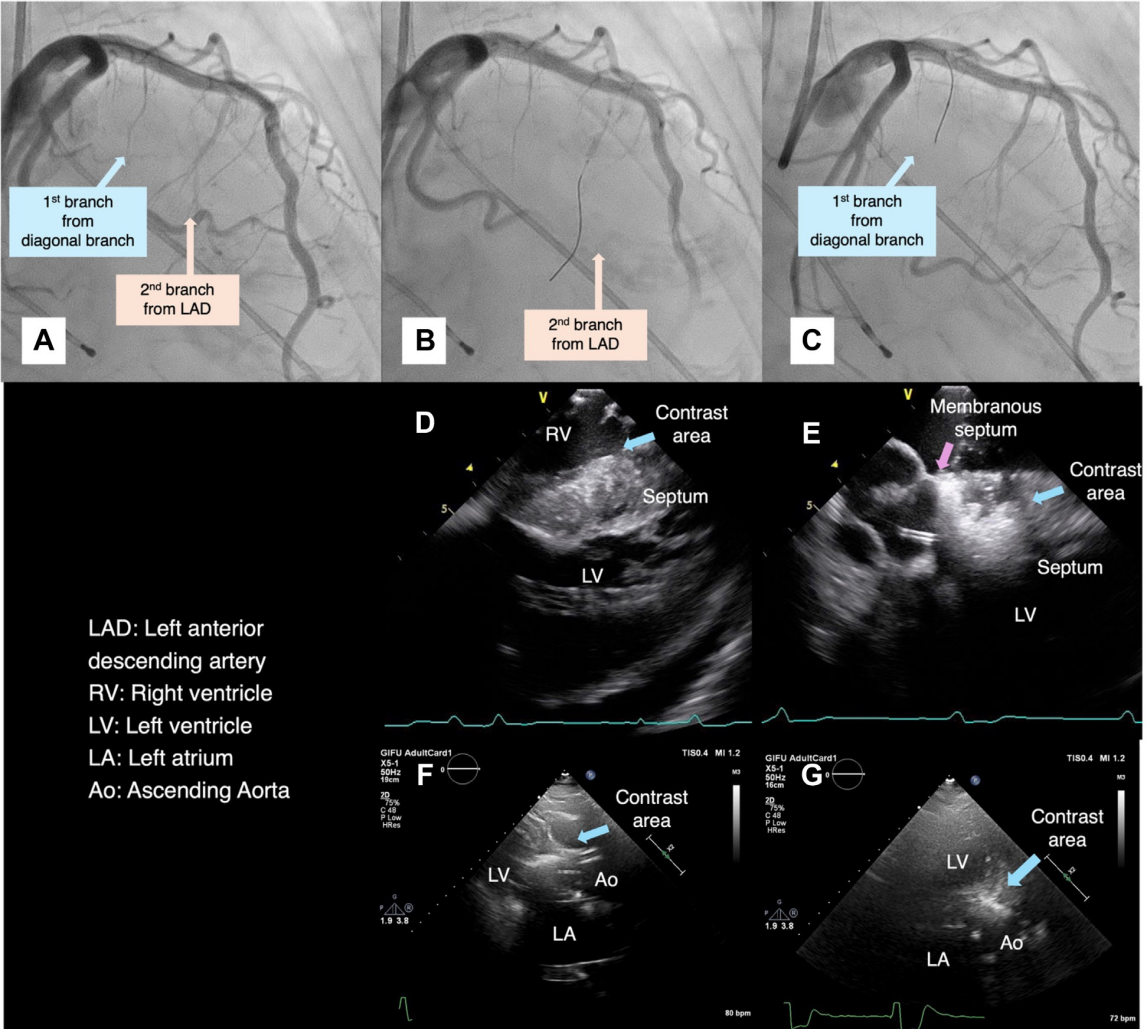
Imaging in Case 3 (A to C) Coronary angiography (A) before ablation, (B) during ballooning to the second branch from the left anterior descending artery (LAD), and (C) during wiring to the first branch from the diagonal branch. (D and E) Jugular vein intracardiac echocardiography showing (D) contrast agent injection to the second branch from the left anterior descending artery and (E) ethanol injection to the first branch from the diagonal branch. High signal intensity is observed up to the membranous septal region. (F and G) Transthoracic echocardiography. (F) Parasternal long-axis view. (G) A 3-chamber view. Compared with (D and E) the intracardiac echocardiography imaging, (F and G) the transthoracic echocardiography imaging was poor. Ao = aorta; LA, left atrium; LV = left ventricle.

A 78-year-old woman with HCM and chronic atrial fibrillation presented with worsening dyspnea (gradient increased from <50 mm Hg to 67 mm Hg). PTSMA was performed using the previous setup. Initially, ICE-guided contrast injection into the major septal branch from the LAD artery opacified the midseptum (Figures 4A, 4B, and 4D, Video 4). Targeting a more proximal area, a contrast agent was injected into a septal branch from the diagonal artery (Figure 4C). After a 1-mL ethanol injection, ICE revealed opacification extending to the membranous septum (Figure 4E, Video 4), resulting in persistent cAVB. A leadless PM was implanted 2 days post PTSMA. The patient was discharged 6 days later with improved symptoms.

### Case 4

**Figure 5:**
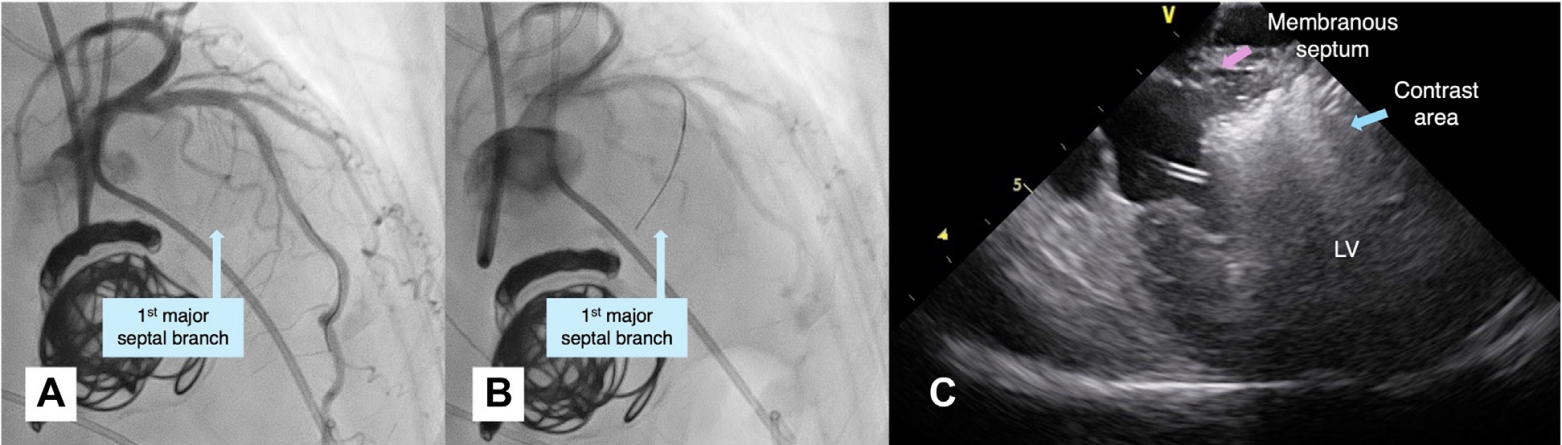
Imaging in Case 4 (A and B) Coronary angiography (A) before ablation and (B) during wiring to the first branch from the left anterior descending artery. (C) Jugular vein intracardiac echocardiography showing an ethanol injection to the first branch from the left anterior descending artery. No high signal intensity is observed in the membranous septal region. LV = left ventricle.

A 79-year-old woman with HOCM underwent PTSMA using the previous setup. Under ICE guidance, a septal branch from the LAD artery ablated (Figures 5A and 5B). Post procedure, her creatine kinase and the MB isoenzyme of creatine kinase levels peaked at 2,777 IU/L and 462 IU/L, respectively, without persistent cAVB. Despite higher cardiac enzyme elevations than in case 3 (Table 1), ICE showed no hyperechoic signal in the membranous septum (Figure 5C, Video 5), a finding suggesting minimal impact on this area. This real-time ICE monitoring provided crucial management information. The patient was discharged 14 days post procedure with improved symptoms.

## Procedural Steps

The following procedure for PTSMA was developed by integrating traditional PTSMA techniques^7^ with novel JV ICE approaches:

1. Prepare necessary equipment:
  • 9-F sheath for ICE catheter (right internal JV)
  • 5-F sheath for LV catheter (right radial artery)
  • 7-F sheath for coronary catheter (right femoral artery)
  • 6-F sheath for intraoperative PM (right femoral vein)
  • 6-F sheath for postoperative PM (left JV)

Note: Use standard vascular access sheaths (eg, Radifocus Introducer II H, Terumo, or equivalent).

**Figure 6:**
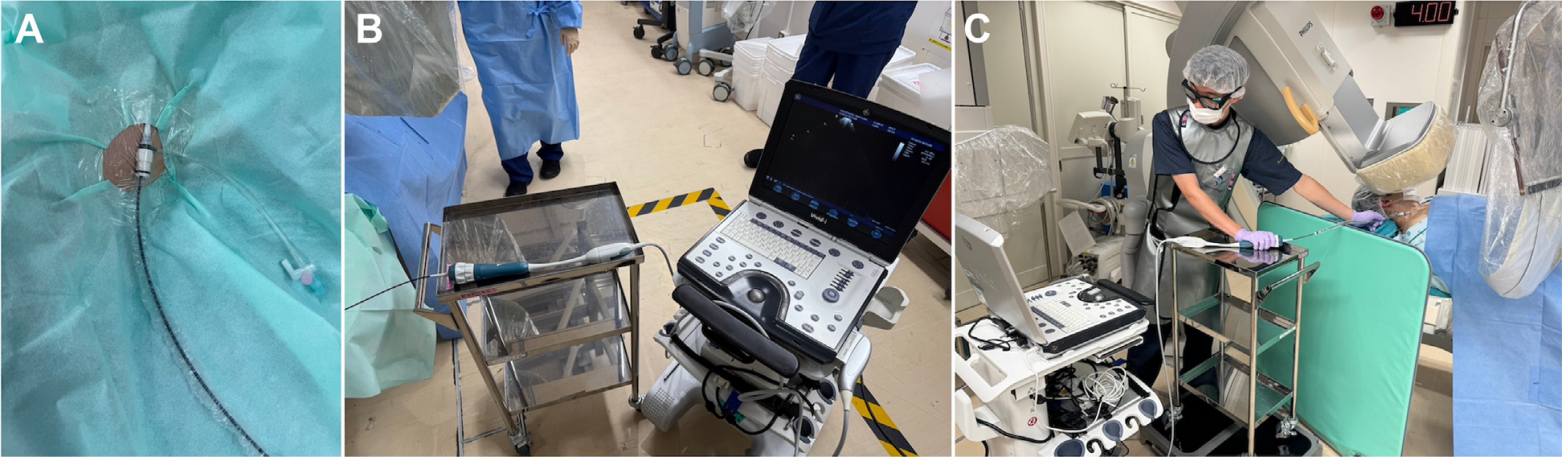
Puncture Site and Intracardiac Echocardiography Setup (A) Intracardiac echocardiography catheter with a sterile sleeve. The use of a sterile sleeve and a transparent seal maintains cleanliness of the puncture site and internal tissues while allowing the echocardiographer to operate in a nonsterile environment. (B) Intracardiac echocardiography device on a table. Placing the intracardiac echocardiography device on a table at the same height as the puncture site enhances stability during the procedure. (C) Echocardiographer operating an intracardiac echocardiography device. Reducing radiation exposure for the echocardiologist is crucial, in addition to maintaining sterile technique. Our institution uses a radiation shield of appropriate size to avoid interference with fluoroscopic equipment.
  b. Manipulate ICE to obtain views:
    i. Long-axis ascending aorta view (Figure 7A):
      • Advance the catheter about 5 cm from the tip of the sheath with clockwise or counterclockwise rotation.
      • Evaluate the ascending aorta before the procedure.

• 6- or 7-F standard guide catheter (eg, Judkins left)
• 4- or 5-F pigtail catheter for LV pressure measurement (eg, mTAKA, Nipro Corp)
• 0.035-inch guidewire
• 0.014-inch coronary guidewire
• Over-the-wire balloon catheter
• Contrast agent: Vitaject (0.2 mL) with low-molecular-weight dextran (2 mL)
• Ethanol (>94%): 2 mL
• Analgesics (eg, morphine, fentanyl)
• Sedatives (eg, propofol, dexmedetomidine)
• Heparin
• Ultrasound system (eg, Vivid Q, GE Healthcare)
2. Anesthesia and vascular access
  a. Induce general anesthesia and manage air with a laryngeal mask and mechanical ventilation.
  b. Establish vascular access and insert the catheters (except ICE).
  c. Administer heparin (maintain an activated clotting time of 200-300 seconds).
  d. Obtain baseline hemodynamics.
3. ICE catheter insertion and initial screening
  a. Insert the ICE catheter using a sterile sleeve, to allow nonsterile manipulation while maintaining internal sterility (Figures 6A to 6C).

**Figure 7:**
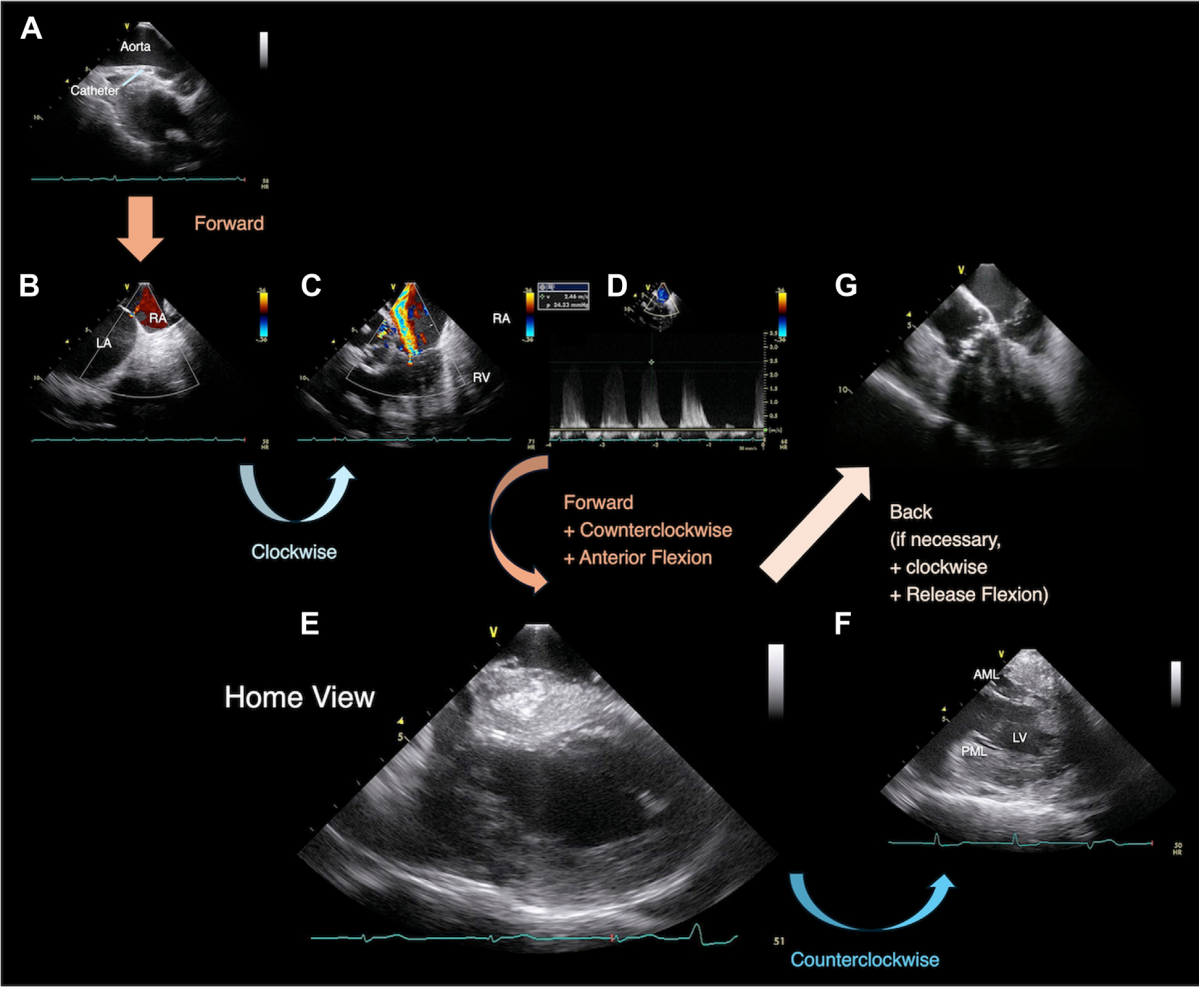
Multiple Views With Jugular Vein Intracardiac Echocardiography Identifying Complications or Hemodynamics (A) Long-axis ascending view. (B) Atrial septum view. There is small atrial septal defect with left-to-right shunt flow. (C) Long-axis tricuspid view. There is mild to moderate tricuspid regurgitation. (D) Pulse Doppler image of tricuspid regurgitation demonstrating the pressure gradient from the right ventricle (RV) to the right atrium (RA) of 24 mm Hg. (E) Long-axis left ventricular view. (F) Long-axis mitral valve view. (G) Long-axis aortic valve view. AML = anterior mitral leaflet; LA = left atrium; LV = left ventricle; PML = posterior mitral leaflet.
    ii. Atrial septum view (Figure 7B):
      • Advance the catheter a few centimeters farther (if necessary, add clockwise rotation).
      • Check for atrial septal defects or a patent foramen ovale.
    iii. Long-axis right ventricular (RV) view (Figure 7C):
      • Rotate the catheter clockwise.
      • Assess for preoperative tricuspid regurgitation and estimate RV systolic pressure (Figure 6D).
    iv. Long-axis LV view (home view) (Figure 7E):
      • Push forward into the right ventricle with anterior flexion and counterclockwise rotation.
      • Evaluate LV contraction, pericardial effusion, and wire position during the procedure.
    v. Long-axis mitral valve view (Figure 7F):
      • Rotate the catheter counterclockwise.
      • Assess the mitral valve complex.
    vi. Long-axis aortic valve view (Figure 7G):
      • Return the catheter clockwise and pull back to the right atrium.
      • Evaluate the aortic valve complex (if necessary).
      • Measure preprocedural aortic valve flow velocity (if necessary).
  c. Perform pretreatment TTE evaluation.
4. Target identification and contrast agent injection
  a. Administer analgesics.
  b. Perform coronary angiography.
  c. Monitor the contrast injection with ICE.
  d. Confirm the target location with ICE, and reassess if needed.
5. Septal ablation and monitoring
  a. Continuously monitor with ICE during the ethanol injection.
  b. Evaluate for complications:
    i. Wall motion abnormalities
    ii. Pericardial effusion
    iii. Mitral valve function changes
    iv. Ventricular septal rupture
    v. Other mechanical complications
6. Postprocedure evaluation and management
  a. Confirm full-cover ablation using ICE and TTE.
    i. Ensure ablation of the portion in contact with accelerated flow on color Doppler 2-dimensional imaging.^8^
  b. Remove the ICE catheter and arterial sheaths.
  c. Insert a temporary PM through the left internal JV.
  d. Remove the intraoperative PM and procedural venous sheaths.
  e. Transfer the patient to the coronary care unit.

**Figure 8:**
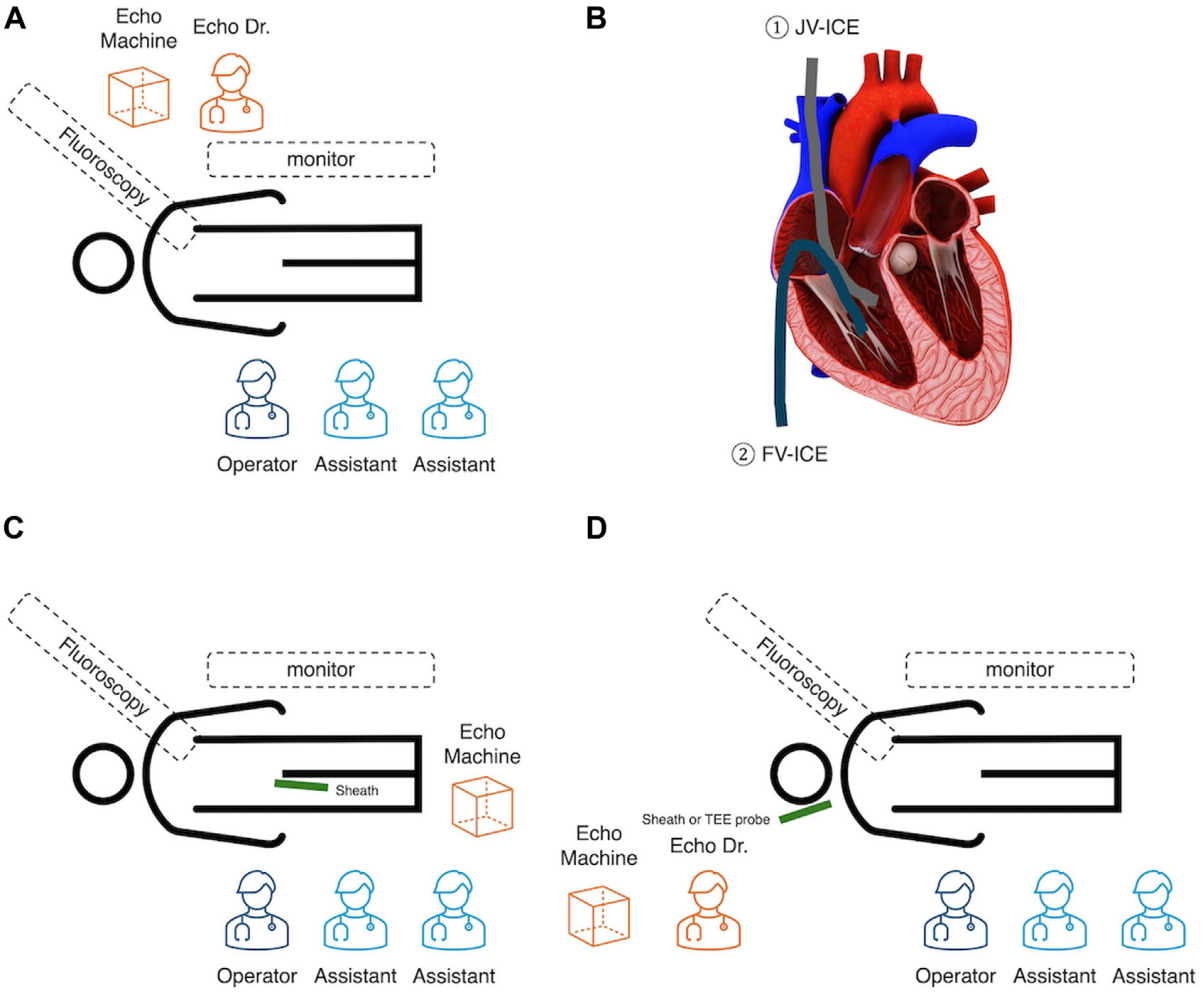
Equipment Setting for Percutaneous Transluminal Septal Myocardial Ablation Using Different Echocardiographic Approaches and ICE Positioning (A) Myocardial contrast echocardiography using a transthoracic echocardiography setup. (B) Comparison of intracardiac echocardiography (ICE) positioning for jugular vein intracardiac echocardiography (JV-ICE) and femoral vein intracardiac echocardiography (FV-ICE). (C) Femoral vein intracardiac echocardiography setup. (D) Jugular vein intracardiac echocardiography or transesophageal echocardiography (TEE) setup. Echo = echocardiography.

JV ICE offers several advantages in PTSMA. Unlike TTE, it provides consistent imaging without fluoroscopy interference^9^ (Figures 8A and 8D) and overcomes the challenges of suboptimal acoustic windows in the supine position (Figures 4F and 4G, Video 6). Compared with TEE, it is less invasive, avoiding intubation risks. It requires less manipulation than FV ICE to access the right ventricle (Figure 8B). JV ICE allows independent echocardiographer operation, freeing the interventionist to focus on the procedure (Figures 8C and 8D). This separation improves workflow and may enhance safety. The dedicated operator can continuously monitor myocardial contrast and ablation, thus enabling quicker complication detection (Figure 6, Video 7). This feature is crucial for precise targeting and size optimization in PTSMA.^4^ The superior imaging with JV ICE may improve identification of target vessels, including those from diagonal or intermediate branches.^3^ However, larger and prospective studies are needed to establish the safety and efficacy of JV ICE compared with other echocardiography techniques in PTSMA.

## Potential Pitfalls

Although JV ICE-guided PTSMA offers potential benefits, it is important to address potential pitfalls. Risks include vascular complications, arrhythmias, cardiac tamponade, embolism, and infections. The technique requires specific skills, presenting a learning curve. An additional consideration is the need for left internal JV access for postoperative PM management, thus increasing total vascular puncture sites.

Mitigation strategies include ultrasound-guided access, careful catheter manipulation, strict sterile technique, and comprehensive training. Optimizing settings and continuous monitoring enhance safety. Although our patients did not experience any complications, and a transcatheter aortic valve implantation study using JV ICE (n = 106) reported no increased risks,^10^ PTSMA differs from transcatheter aortic valve implantation, and these results require cautious interpretation. A stepwise approach and collaboration with experienced centers can help overcome initial challenges.

## Conclusions

Our case series demonstrates the feasibility and potential advantages of JV ICE guided PTSMA. By addressing limitations of TTE and FV ICE, it offers enhanced visualization and guidance for PTSMA. This technique could improve PTSMA safety and efficacy. Prospective studies with long-term follow-up are essential to evaluate the role of JV ICE in HOCM treatment fully.

## Funding Support and Author Disclosures

Dr Yagasaki has received MitraClip proctorship fees from Abbott Medical Japan that are not directly related to the content of this paper. All other authors have reported that they have no relationships relevant to the contents of this paper to disclose.

## Appendix

Video 1 ICE Imaging of Contrast Injection in Case 1

Video 2 ICE Imaging of Ethanol Injection in Case 1

Video 3 ICE Imaging of Contrast and Ethanol Injection in Case 2

Video 4 ICE Imaging of Contrast and Ethanol Injection in Case 3

Video 5 ICE Imaging of Ethanol Injection in Case 4

Video 6 TTE Imaging After Ethanol Injection in Case 3

Video 7 ICE Imaging for Detecting Complications and Assessing Hemodynamics

## References

[1] Writing Committee Members, Ommen S.R., Ho C.Y. (2024). 2024 AHA/ACC/AMSSM/HRS/PACES/SCMR guideline for the management of hypertrophic cardiomyopathy: a report of the American Heart Association/American College of Cardiology Joint Committee on Clinical Practice Guidelines. J Am Coll Cardiol.

[2] Maron M.S., Olivotto I., Zenovich A.G. (2006). Hypertrophic cardiomyopathy is predominantly a disease of left ventricular outflow tract obstruction. Circulation.

[3] Faber L., Seggewiss H., Gleichmann U. (1998). Percutaneous transluminal septal myocardial ablation in hypertrophic obstructive cardiomyopathy: results with respect to intraprocedural myocardial contrast echocardiography. Circulation.

[4] Monakier D., Woo A., Puri T. (2004). Usefulness of myocardial contrast echocardiographic quantification of risk area for predicting postprocedural complications in patients undergoing septal ethanol ablation for obstructive hypertrophic cardiomyopathy. Am J Cardiol.

[5] Pedone C., Vijayakumar M., Ligthart J.M.L. (2005). Intracardiac echocardiography guidance during percutaneous transluminal septal myocardial ablation in patients with obstructive hypertrophic cardiomyopathy. Int J Cardiovasc Intervent.

[6] Vaina S., Ligthart J., Vijayakumar M. (2006). Intracardiac echocardiography during interventional procedures. EuroIntervention.

[7] Maekawa Y., Takamisawa I., Takano H., Takayama M. (2022). Percutaneous transluminal septal myocardial ablation: past, present, and future. J Cardiol.

[8] Holmes D.R., Valeti U.S., Nishimura R.A. (2006). Alcohol septal ablation for hypertrophic obstructive cardiomyopathy: a systematic review of published studies. J Interv Cardiol.

[9] Yagasaki H., Goto Y., Mori Y., Noda T. (2018). Transcatheter aortic valve replacement with intracardiac echocardiography from the right internal jugular vein. Cardiovasc Diagn Ther.

[10] Ishizu K., Shirai S., Miyawaki N. (2024). Impact of transjugular intracardiac echocardiography-guided self-expandable transcatheter aortic valve implantation on reduction of conduction disturbances. Circ Cardiovasc Interv.

